# Author Correction: Spatial and temporal fluctuations in COVID-19 fatality rates in Brazilian hospitals

**DOI:** 10.1038/s41591-022-01939-4

**Published:** 2022-07-14

**Authors:** Andrea Brizzi, Charles Whittaker, Luciana M. S. Servo, Iwona Hawryluk, Carlos A. Prete, William M. de Souza, Renato S. Aguiar, Leonardo J. T. Araujo, Leonardo S. Bastos, Alexandra Blenkinsop, Lewis F. Buss, Darlan Candido, Marcia C. Castro, Silvia F. Costa, Julio Croda, Andreza Aruska de Souza Santos, Christopher Dye, Seth Flaxman, Paula L. C. Fonseca, Victor E. V. Geddes, Bernardo Gutierrez, Philippe Lemey, Anna S. Levin, Thomas Mellan, Diego M. Bonfim, Xenia Miscouridou, Swapnil Mishra, Mélodie Monod, Filipe R. R. Moreira, Bruce Nelson, Rafael H. M. Pereira, Otavio Ranzani, Ricardo P. Schnekenberg, Elizaveta Semenova, Raphael Sonabend, Renan P. Souza, Xiaoyue Xi, Ester C. Sabino, Nuno R. Faria, Samir Bhatt, Oliver Ratmann

**Affiliations:** 1 Department of Mathematics, Imperial College London, London, UK; 2 MRC Centre for Global Infectious Disease Analysis, Jameel Institute, School of Public Health, Imperial College London, London, UK; 3 Institute for Applied Economic Research, IPEA, Brasília, Brazil; 4 Departamento de Engenharia de Sistemas Eletrônicos, Escola Politécnica, Universidade de São Paulo, São Paulo, Brazil; 5 World Reference Center for Emerging Viruses and Arboviruses and Department of Microbiology and Immunology, University of Texas Medical Branch, Galveston TX, USA; 6 Departamento de Genética, Ecologia e Evolução, Instituto de Ciências Biológicas, Universidade Federal de Minas Gerais, Belo Horizonte, Brazil; 7 Instituto D’Or de Pesquisa e Ensino (IDOR), Rio de Janeiro, Brazil; 8 Laboratory of Quantitative Pathology, Center of Pathology, Adolfo Lutz Institute, São Paulo, Brazil; 9 Programa de Computação Científica, Fundação Oswaldo Cruz, Rio de Janeiro, Brazil; 10 Departamento de Moléstias Infecciosas e Parasitárias e Instituto de Medicina Tropical da Faculdade de Medicina, Universidade de São Paulo, São Paulo, Brazil; 11 Department of Zoology, University of Oxford, Oxford, UK; 12 Department of Global Health and Population, Harvard T. H. Chan School of Public Health, Boston MA, USA; 13 Department of Epidemiology of Microbial Diseases, Yale School of Public Health, New Haven CT, USA; 14 Latin American Centre, University of Oxford, Oxford, UK; 15 Department of Computer Science, University of Oxford, Oxford, UK; 16 Department of Microbiology, Immunology and Transplantation, Rega Institute, KU Leuven – University of Leuven, Leuven, Belgium; 17 Section of Epidemiology, School of Public Health, University of Copenhagen, Copenhagen, Denmark; 18 Departamento de Genética, Instituto de Biologia, Universidade Federal do Rio de Janeiro, Rio de Janeiro, Brazil; 19 Environmental Dynamics, INPA, National Institute for Amazon Research, Manaus, Brazil; 20 Barcelona Institute for Global Health, ISGlobal, Barcelona, Spain; 21 Nuffield Department of Clinical Neurosciences, University of Oxford, Oxford, UK; 22 Department of Infectious Disease Epidemiology, Imperial College London, London, UK

**Keywords:** Health care, Developing world

Correction to: *Nature Medicine* 10.1038/s41591-022-01807-1, published online 10 May 2022.

In the version of this article initially published, there was an error in the spelling of Raphael Sonabend’s surname (originally appearing as Sonnabend). The error has been corrected in the HTML and PDF versions of the article.

